# Emergency Clinician Experiences Using a Standardized Communication Tool for Cardiac Arrest

**DOI:** 10.7759/cureus.9759

**Published:** 2020-08-15

**Authors:** Casey Carr, Joshua Hardy, Becca Scharf, Matthew Levy

**Affiliations:** 1 Department of Emergency Medicine, University of Florida - Shands, Gainesville, USA; 2 School of Medicine, Georgetown University, District of Columbia, USA; 3 Department of Fire and Rescue Services, Howard County Department of Fire and Rescue Services, Marriottsville, USA; 4 Department of Emergency Medicine, Johns Hopkins University School of Medicine, Baltimore, USA; 5 Office of the Medical Director, Howard County Department of Fire and Rescue Services, Marriottsville, USA

**Keywords:** out of hospital cardiac arrest, interdisciplinary communication, post cardiac arrest care

## Abstract

Introduction

Sudden cardiac arrest remains a common and critical disease burden. As post-cardiac arrest care grows in complexity, communication between pre-hospital providers, emergency department personnel, and hospital consultants is increasingly important.

Methods

This study evaluated the use of a standard handoff tool between pre-hospital personnel and hospital staff, including emergency medical services (EMS), emergency department nurses, physicians, and cardiologists. Personnel were surveyed regarding attitudes surrounding the important aspects of cardiac arrest care, challenges faced, and preference of handoff mechanism.

Results

Most of the survey respondents (58, 76%) found that the initial rhythm was the most important factor in post-cardiac arrest care, followed by the presence of bystander cardiopulmonary resuscitation (CPR; 55, 72%) and the presence of ST-elevation on initial electrocardiogram (46, 61%). Both emergency physicians (7, 63%), as well as cardiologists (3, 100%), preferred to have this tool performed over radio prior to arrival in the emergency department.

Conclusion

The importance given to various post-cardiac arrest factors varied amongst specialty and clinical background; however, all agreed on common features such as the initial rhythm, electrocardiogram (ECG) morphology, and the presence or absence of bystander CPR. Additionally, the timing and structure of how this information is delivered were further elucidated. This data will guide future handoff methods between specialties managing patients after cardiac arrest.

## Introduction

Sudden cardiac arrest remains a critical and common problem; current estimates suggest that there are over 300,000 episodes of sudden cardiac arrest in the United States annually [1]. Despite ongoing advances in cardiac arrest care, an out-of-hospital cardiac arrest has had essentially unchanged mortality outcomes for the past 20 years with a remarkable degree of regional variability [2-3]. Many of the ongoing changes in the paradigm of cardiac arrest management focus on the post-arrest period [4-5].

As post-cardiac-arrest care continues to gain in complexity and coordination, the handoff of critical information between pre-hospital EMS clinicians and in-hospital personnel has heightened in importance. Recommendations from cardiology professional societies incorporate information gathered during prehospital care into the timing of decisions regarding post-arrest coronary angiography [6]. This information includes whether or not the arrest was witnessed, presence of bystander CPR, first rhythm, on-scene time, and length of resuscitation, which are obtained from prehospital responders. Of additional importance is the communication with family members by pre-hospital providers. Frequently, the only history available is during this time, and the presence of terminal disease or known atherosclerotic coronary artery disease can make various etiologies of cardiac arrest more likely.

Patients who have survived to hospital admission after cardiac arrest seem to have better outcomes after coronary angiography, even in the absence of ST-elevation on electrocardiogram (ECG) [7]. In light of this, the American College of Cardiology developed an algorithm using resuscitative prognostic features such as presenting rhythm, bystander CPR, and whether or not the cardiac arrest was witnessed [6]. While current recommendations utilize multiple resuscitative prognostic factors, there is no individual weight. Different clinical teams may consider various factors more important than another, for instance, pre-hospital providers may emphasize the importance of bystander CPR, while inpatient cardiology may view arrest rhythm as the most important.

Miscommunication between teams has been identified as a critical source of medical error, and this is often exacerbated in the emergent setting [8]. Which of the specific clinical elements that are lost during inter-disciplinary teams varies based upon the perception of what is important to each discipline. The exact degree of difference in the perception of the importance of information, such as between emergency medicine and cardiology, has yet to be defined in the literature. Standardized handoff tools have been developed for change of care between in-hospital staff [9]; however, an analogous handoff tool, containing critical post-cardiac arrest information, has yet to be developed for post-arrest patients.

## Materials and methods

Study setting and design

This was a convenience sample study evaluating the communication between resuscitation team members and interventional cardiologists between April 2018 and September 2018. The setting was a 48-bed community hospital emergency department that has emergency percutaneous cutaneous coronary intervention (PCI) capability and an annual volume of 77,000 patients. Howard County, Maryland, has a population of approximately 300,000. The Howard County Department of Fire and Rescue services transport approximately 18,500 patients annually. The cardiac catheterization lab performed 92 emergent PCI cases in 2019.

When a return of spontaneous circulation after an out-of-hospital cardiac arrest occurred, the paramedics would notify the receiving emergency department via radio. On arrival, a pre-made sticker with four questions was placed on the patients’ chart and the resuscitation team members would fill this form. The questions consisted of: Witnessed arrest (Y/N), Bystander CPR (Y/N), Initial rhythm VT/VF PEA/Asystole, and Arrest time. Nursing staff would document the results of this form in the electronic medical system. This note was then shared with all members of the resuscitation team and interventional cardiologists.

Study population and survey instrument

All emergency physicians, cardiologists, and emergency nurses who are involved in the care of out-of-hospital cardiac arrest patients were invited to complete a 25-question survey. Survey questions were based on those that were anecdotally reported as common in occurrence and clinically important by members of the study team. Individual resuscitation team members and interventional cardiologists were asked to pick the four most important data elements from a list of choices that they would want to know in a post-out-of-hospital cardiac arrest with the return to spontaneous circulation case, as well as the challenges faced based on the experience of the clinicians in their field regarding these cases.

Outcome measure

The primary outcome was the frequency and percentage of preferred post-cardiac arrest data by respondents. The secondary outcome was the perception of difficult aspects of post-cardiac arrest care stratified by personnel background.

Data analysis and statistics

Data was collected and compiled with the use of survey software (Survey Monkey Inc., San Mateo, California) and exported to Microsoft® Excel (Microsoft Corporation, Redmond, Washington) for analysis. Descriptive statistics were performed using Microsoft® Excel (Microsoft Corporation). Data were abstracted and analyzed by two reviewers, who were not independent of each other.

Ethics statement

The study was approved by the Institutional Review Board of Johns Hopkins University.

## Results

**Figure 1:**
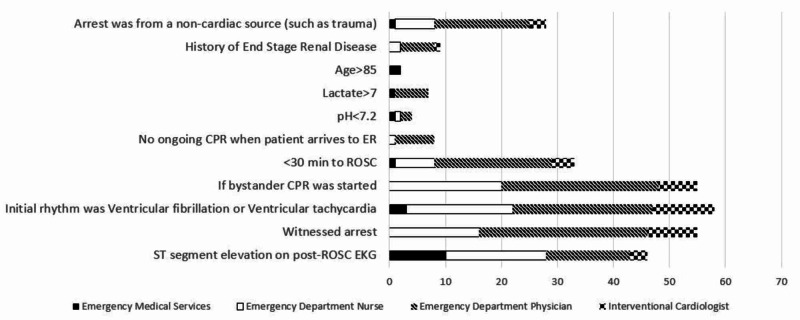
Post-cardiac feature preference by clinical role

Of the 200 individuals who received the survey, a total of 76 (38.0%) completed the survey. The results of the survey are summarized in Table 1. Through the survey, it was determined that 58 (76%) respondents wanted to know if the initial rhythm was ventricular tachycardia/ventricular fibrillation, 55 (72%) respondents wanted to know if both the cardiac arrest was witnessed and if bystander cardiopulmonary resuscitation was started before emergency medical services arrived, and 46 (61%) respondents wanted to know if there was ST-elevation on post-resuscitation of spontaneous circulation on the ECG. Figure 1 provides a further breakdown of responses by personnel type. Three of the top four choices that resuscitation team members and interventional cardiologists selected were most important to know in a post-return of the spontaneous case were included in the standardized pre-hospital communication tool.

**Table 1:** Post-cardiac arrest features preference results OHCA: out-of-hospital cardiac arrest; ROSC: return of spontaneous circulation

OHCA-ROSC Data Points	Preference Frequency (%)
Initial rhythm was ventricular fibrillation or ventricular tachycardia	58 (76.32%)
Patient experienced a witnessed arrest	55 (72.37%)
Bystander CPR initiated before EMS arrived	55 (72.37%)
Presence of ST-elevation on post-ROSC electrocardiogram	46 (60.53%)
Patient achieved a return to spontaneous circulation in less than 30 minutes after cardiac arrest	33 (43.42%)
Arrest was from a non-cardiac source	28 (36.84%)
History of end-stage renal disease	9 (11.84%)
Ongoing CPR in the emergency department	8 (10.53%)
Lactate greater than 7	7 (9.21%)
pH less than 7.2	4 (5.26%)
Patient older than 85 years old	2 (2.63%)

Other notable data points that resuscitation team members and interventional cardiologists felt were most important include were: if the patient returns to spontaneous circulation less than 30 minutes after cardiac arrest (33; 43%), if the patient had a non-cardiac etiology of arrest (28; 37%), if the patient had a history of end-stage renal disease (9; 12%), if there was ongoing cardiopulmonary resuscitation in the emergency department (8; 11%), if the patient had lactate greater than 7 mm/L (7, 9%), if the patient had a pH less than 7.2 (4; 5%), and if the patient was older than 85 years old (2, 3%).

Regarding the timing of communication, the respondents from the emergency medical services, 22 (85%) wanted to relay the standard pre-hospital communication tool data on arrival to the emergency department as opposed to 24 (60%) of emergency department registered nurses, seven (63%) of emergency physicians, and three (100%) of interventional cardiologists wanting to have the standardized pre-hospital communication data relayed via radio to the emergency department.

**Figure 2:**
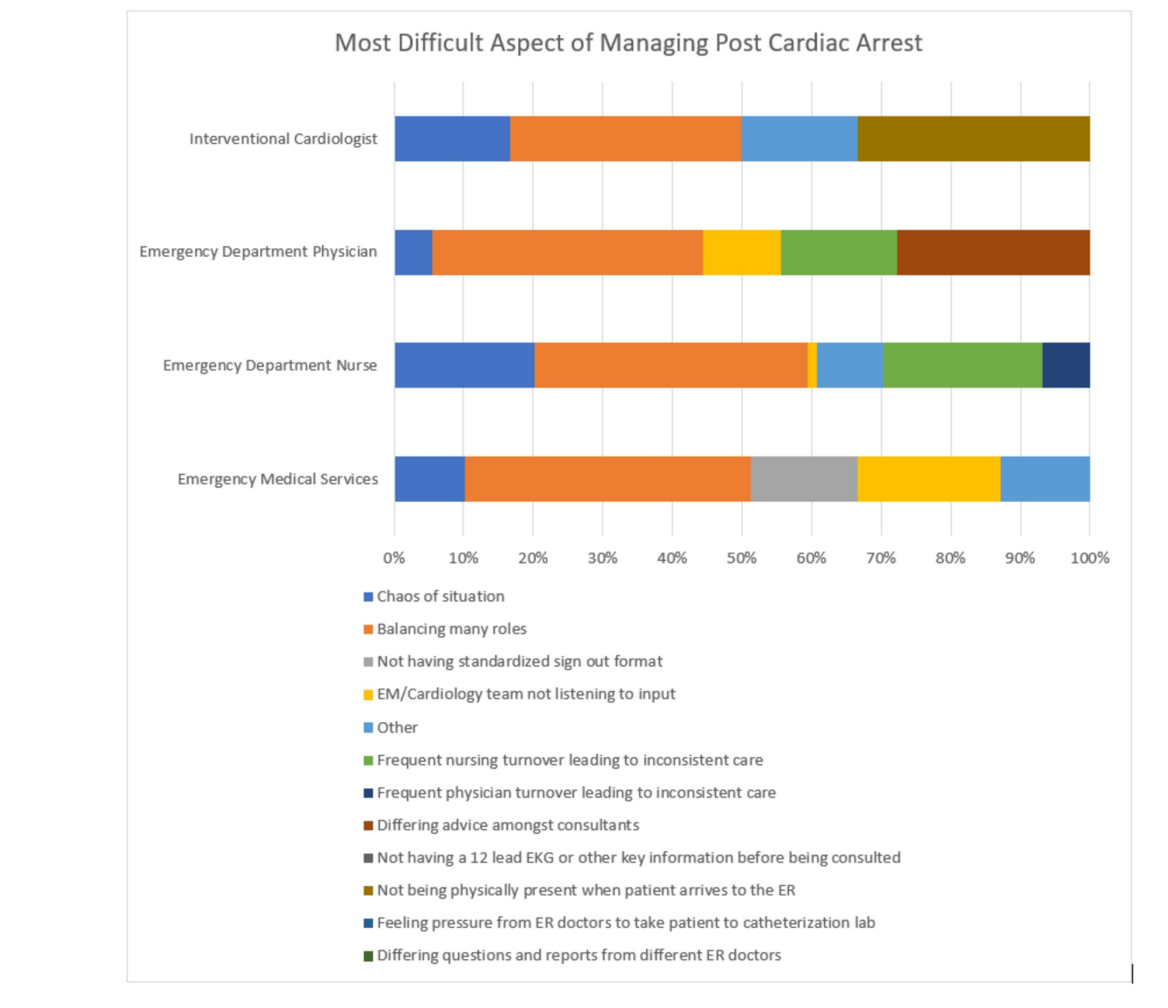
Difficult aspects of managing post-arrest care by clinical role

Figure 2 lists the options and results regarding the perceived difficult aspects of managing post-cardiac arrest care.

From the perspective of the emergency medical service clinicians, 16 (70%) of respondents stated that balancing many roles, including scene safety, patient care, supporting the patient’s family, transferring data via radio to the emergency department, and transporting the patient was the most difficult part. In addition, eight (35%) of emergency medical service respondents felt that the emergency department was not listening to their input regarding the case and six (26%) of respondents stated that not having a standardized checklist or protocol to use when communicating key information on out-of-hospital cardiac arrest cases with a return to spontaneous circulation to the emergency department was the most difficult part of managing the case.

From the perspective of registered nurses, 29 (75%) of respondents stated that balancing roles, including EMS report, physician orders, patient care, and talking with family, was the most difficult part of managing post-return of spontaneous circulation cases. Also, 33 (94%) of registered nurses who were surveyed also stated that the standardized pre-hospital communication tool and electronic medical note that was created was very easy, easy, or neutral to fill out.

Emergency department physicians stated that balancing roles, including the EMS report, nurse interaction, patient care, interacting with family members, and cardiology consult was the most difficult part of managing post-return of spontaneous cases (7, 64%) and noted that variability in advice and answers from cardiology consults (5, 45%) was the difficult part of managing the post-return to spontaneous circulation case.

Interventional cardiologists also felt that balancing roles, including assembling the interventional cardiology team, receiving the medical history of the patient from the emergency department physician, and reviewing the electrocardiogram and patent’s chart, was the most difficult part of managing post-return of spontaneous circulation cases (2, 67%). They also commented on the difficulty of not being present in the emergency department when the patient arrives and the case is presented (2, 67%). Two (66%) of interventional cardiologists that responded to the survey stated that the standardized pre-hospital communication tool would be helpful in providing a scripted case presentation of out-of-hospital cardiac arrest with the return of spontaneous circulation cases.

## Discussion

A paucity of data exists regarding the attitudes of clinical teams regarding individual post-resuscitative factors. The optimal manner by which this information is relayed, as well as what information is most important to treating interventional cardiologists, and when this information is given, has yet to be defined. Additionally, each interdisciplinary team faces challenges unique to their setting and field, making communication between teams individually challenging, for instance, when should clinical post-cardiac arrest information be reported? Knowledge of these parameters, including which post-arrest prognostic factor to report, when this information should be reported, and how to best disseminate this information, is of critical importance when making interdisciplinary patient-centered clinical decisions.

This survey highlights the differences in attitudes amongst various clinical teams surrounding post-resuscitative communication and clinical management. Specifically, pre-hospital clinicians, emergency department nurses, emergency physicians, and interventional cardiologists were included in a cross-sectional survey. This study attempted to understand attitudes about post-ROSC management and views about a recently implemented communication tool. These results demonstrated that in regard to post resuscitative prognostic features, there was a general alignment among all teams. However, interventional cardiologists felt like the most important feature was initial arrest rhythm and emergency physicians felt like whether the arrest was witnessed or not was the most important. All respondents to the survey felt like post-ROSC data was either extremely important or very important in determining patient management. Among all teams, the most difficult component of management out-of-hospital cardiac arrest was commonly managing many roles, the chaos of the situation, and obtaining a medical history and arrest time. In-hospital teams preferred receiving post-ROSC data via radio prior to arrival and all interventional cardiologists were either not able to gather post-ROSC data or not gather it in an organized format.

As the management of post-cardiac arrest patients continues to evolve, the need for coordination of streamlined communications between interdisciplinary teams is essential. In contemporary post-cardiac arrest management, cardiac catheterization continues to be of ongoing interest. While there have been conflicting literature on timing and efficacy, current guidelines recommend using resuscitative prognostic indicators as a decision point for whether or not to proceed to cardiac catheterization [6,10]. Given these recommendations, ensuring that accurate and coherent exchange of information between multiple clinical teams is paramount. To our knowledge, no prior study has examined the attitudes regarding post-ROSC data between resuscitation team members and the communication that exists between teams. These results demonstrate that while a small amount of disagreement exists, most team members agree on what post-ROSC data is clinically meaningful. However, the means by which the data is reported, and when to report them, is different between teams.

This study helps highlight which data each team feels should be reported and that in-hospital resuscitation teams prefer to hear the report via radio prior to arrival. Additionally, all team members felt like balancing multiple roles was the most difficult aspect of post-ROSC management. It stands to reason that given this finding, standardized handoff tools would be of critical importance to reduce the burden of task-switching. Lastly, interventional cardiologists felt like obtaining post-ROSC data was either not possible or disorganized, which again speaks to the utility of a uniform post-ROSC handoff tool and universal language. Bringing all resuscitative team members’ interests into alignment promotes patient safety, likely increases adherence with best-practice and professional society recommendations, and increases ease of transition of care.

There were limitations to this study. The data derived were survey data, and given the recipients were prone to turnover and had a variable response rate, could be a potential source of bias. The data was cross-sectional and, therefore, the change in the attitudes of clinicians could not be captured. Additionally, the sample size was small. This study was also limited by sample size and interdisciplinary participation, specifically amongst the small number interventional cardiologists. The survey was performed in the setting of an ongoing quality improvement project, which by itself may have impacted attitudes among resuscitation team members.

## Conclusions

We have demonstrated the differing attitudes regarding post-ROSC management, data communication, and timing of data reporting amongst an interdisciplinary resuscitation group at a community hospital. These differing attitudes, specifically among timing, data to report, and the importance of organization, demonstrate the need for continued communication training and tool development.
